# The Emerging Role of Amino Acids of the Brain Microenvironment in the Process of Metastasis Formation

**DOI:** 10.3390/cancers13122891

**Published:** 2021-06-09

**Authors:** Francesca Cutruzzolà, Amani Bouzidi, Francesca Romana Liberati, Sharon Spizzichino, Giovanna Boumis, Alberto Macone, Serena Rinaldo, Giorgio Giardina, Alessio Paone

**Affiliations:** Laboratory Affiliated to Istituto Pasteur Italia, Department of Biochemical Sciences A. Rossi Fanelli, Sapienza University of Rome, Piazzale Aldo Moro 5, 00185 Rome, Italy; francesca.cutruzzola@uniroma1.it (F.C.); amani.bouzidi@uniroma1.it (A.B.); francescaromana.liberati@uniroma1.it (F.R.L.); sharon.spizzichino@uniroma1.it (S.S.); giovanna.boumis@uniroma1.it (G.B.); alberto.macone@uniroma1.it (A.M.); serena.rinaldo@uniroma1.it (S.R.); giorgio.giardina@uniroma1.it (G.G.)

**Keywords:** metastasis, brain microenvironment, organotropism, neurotransmitters, amino acids, metabolism, endothelial cells of the blood–brain barrier

## Abstract

**Simple Summary:**

Why some cancers choose to form metastases in one organ rather than another is still largely unknown. In this review, we summarized the available information on the possible mechanisms controlling this choice. In particular, we tried to understand how some molecules, especially amino acids, released into the environment outside the cells, participate in selecting the brain as a target organ for the formation of metastases by specific types of aggressive tumors such as melanoma, breast, and lung cancer.

**Abstract:**

Brain metastases are the most severe clinical manifestation of aggressive tumors. Melanoma, breast, and lung cancers are the types that prefer the brain as a site of metastasis formation, even if the reasons for this phenomenon still remain to be clarified. One of the main characteristics that makes a cancer cell able to form metastases in the brain is the ability to interact with the endothelial cells of the microvasculature, cross the blood–brain barrier, and metabolically adapt to the nutrients available in the new microenvironment. In this review, we analyzed what makes the brain a suitable site for the development of metastases and how this microenvironment, through the continuous release of neurotransmitters and amino acids in the extracellular milieu, is able to support the metabolic needs of metastasizing cells. We also suggested a possible role for amino acids released by the brain through the endothelial cells of the blood–brain barrier into the bloodstream in triggering the process of extravasation/invasion of the brain parenchyma.

## 1. Introduction

The ability to create metastases in areas distant from the site of the primary tumor is the key feature that determines the malignancy of a tumor. The metastatic process is divided into various steps: initially, the cells detach from the basement membrane of the primary tumor, then enter into the circulation. In the third step, the cells stop inside the small vessels of the target organ and extravasate to invade the organ parenchyma. Finally, the cells adapt to the new microenvironment and grow, giving rise to the actual metastasis.

The first theory on metastasis formation was formulated by Steven Paget [1] and called “seed and soil”. In his analysis, he argued that tumors do not form metastases in random organs, but rather that the host organ (soil) supports the growth of tumor cells (seed), underlying the importance of the target organ microenvironment in metastasis development [1]. After the formulation of Paget’s theory, it was demonstrated that the adhesion process of metastasizing cells on the endothelial cells of the microvasculature of the target organ was a key event in the process of organ selection, indicating also that the migration of metastasizing cells from the bloodstream to the organ parenchyma is another fundamental step [2].

Paget’s theory was challenged by another one which hypothesized that dissemination of cancer cells in specific organs was linked to physical mechanisms, such as blood flow or lymphatic drainage. The first organ encountered by chance on the way of cancer cells along these pathways was the one showing the highest degree of metastases [3]. Currently, the two theories are considered complementary and in apparent contrast with a third hypothesis called “chemotaxis–metastasis theory” according to which some organs could produce factors with a chemoattractant function, responsible for the migration of cancer cells to the future site of metastasis. As an example, it has been shown that the interaction between the chemokine receptors CXCR4 and CCR7, overexpressed on breast cancer and melanoma cells, and their relative ligands (CXCL12/SDF-1α and CCL21/6Ckine), in turn overproduced on the endothelium of some of the main target organs such as lung, liver, lymph nodes, and bone marrow, participate in the metastatic process [4].

Metastases forming in the brain are among the most dangerous ones, due to the delicacy of the organ and the link to vital functions that often makes the surgery option difficult or impossible. Epidemiological studies clearly show that lung (19–40%), breast (5–20%), and melanoma (7–15%) are the types of cancer that mainly form brain metastases [5]. Regardless of the origin of the primary tumor, patients with cerebral metastasis have a poor prognosis with a median survival between 2 and 12 months [6]. Conventional therapies include surgery, radiotherapy, chemotherapy, and immunotherapy but with rather modest results, and the 3-year survival rate of these patients is only 4.8% [7,8].

In this review, we focused our attention on the role of selected amino acids such as serine, glycine, glutamate, and glutamine in affecting brain tropism of cancer cells. We discuss how the trafficking and homeostasis of these amino acids in the brain shape the microenvironment which then supports metastasizing cells. Furthermore, we describe the metabolic adaptations that allow cancer cells to grow using the amino acids released in the brain extracellular fluid. Finally, we propose a novel perspective on the involvement of these amino acids in the mechanism that may guide cancer cells invasion in the brain parenchyma.

## 2. The Endothelial Cells of the Blood–Brain Barrier and the Brain Microenvironment

Due to the delicacy of the organ and its peculiar physiology, the brain is defended from the “outside world” by a particular system placed right at the level of the cerebral blood vessels called blood–brain barrier (BBB). Unlike the more common vessels, those of the BBB are not fenestrated, and the endothelial cells are connected by complex tight junctions formed by many proteins including claudins, occludins, cadherins, connexins etc. These cells are also characterized by a low number of pores on the membrane and show reduced pinocytosis, to tightly control the entry of unwanted molecules and cells [9]. Endothelial cells of the BBB are embedded in a peculiar basal lamina to which other cell types such as pericytes and astrocytes also attach. This specific extracellular matrix is formed mainly by glycoproteins that can be cleaved, playing a fundamental role in specific physiological or pathological processes, including immune and cancer cells invasion [10,11]. A lymphatic-like system called the “glymphatic clearance pathway” has recently been confirmed in the brain [12,13]. Although an implication of this system in different brain pathologies has been hypothesized [14], at the moment there is no evidence of an involvement in metastatic processes.

Inside the brain, cells communicate by releasing and continuously reabsorbing large quantities of neurotransmitters in response to specific stimuli that trigger an electrical signal. In addition, neurons are not able to synthesize several metabolic intermediates which are in turn produced by the surrounding cells, released into the extracellular fluid and absorbed by the neurons. These metabolites, together with specific growth factors and the neurotransmitters create the extremely peculiar brain extracellular environment, also playing a key role in metastasis formation, as detailed below. Melanoma cells, for example, have been initially described to be attracted by neurotrophins like nerve growth factor (NGF) and neurotrophin2 [15]. In addition neurotransmitters have been described to induce chemo-attraction of breast cancer cells. A clear chemo-kinetic effect on MDA-MB-468 breast cancer cells is given by met-enkephalin, substance P, bombesin, dopamine, and norepinephrine, the latter showing a real chemo-attracting effect on both breast and colon cancer cell lines, possibly due to the activation of beta-adrenoreceptors on cancer cells [16,17]. However, the mechanism by which brain-derived neurotransmitters are released by the endothelial cells of the BBB and attract circulating cancer cell remains to be clarified.

The metabolic adaptation to microenvironmental nutrients of the target organ by cancer cells deriving from breast, lung, melanoma, and pancreatic cancers is fairly complex, often involving not only a reprogramming of their own metabolism, but also of that of the other cells present in the microenvironment, by stimulating the production and secretion of molecules necessary for the cancer cell’s metabolism [18,19]. Non-essential extracellular amino acids, like serine and glutamine, play a fundamental role in supporting the growth of cancer cells. It has been shown that cancer cells from different tissues base a large part of their energy production and anabolic metabolism on amino acids present in the extracellular environment, often becoming essentially auxotrophic for selected amino acids, even if the metabolic apparatus for their de novo synthesis is fully functional. As an example, HCT116 colon cancer cells with an intact de novo serine synthesis pathway (SSP) (see below) slowly form a tumor mass if injected in a mouse fed with a serine-/glycine-free diet with respect to the same cells injected in a mouse fed with a normal control diet [20,21]. It is therefore possible to hypothesize that, when specific cell types metastasize in the brain, this organ’s microenvironment meets the essential needs of the invading cells, after they have adapted to the available nutrients such as amino acids and neurotransmitters normally released in the extracellular fluid.

A more detailed analysis of the homeostasis of selected amino acids in the brain microenvironment is presented below.

## 3. Serine/Glycine and Glutamate/Glutamine in Normal Brain

Although it is reported that the concentration of amino acids within human brain extracellular fluid (BEF) is lower than in plasma, a complete and solid quantification is not currently available [22]. Fundamental information comes from the cerebrospinal fluid analysis of healthy patients used as control in a study on Alzheimer’s disease [23]; the results obtained in this work are similar to data obtained from the analysis of mice brains [24]. Data from both papers indicate that only few amino acids are present in a relevant amount in the cerebral extracellular fluid, including some very interesting ones such as serine, glycine, valine, glutamine, and glutamate, shown to sustain the growth of tumor cells or involved in the process of cell migration from the blood circulation into the brain parenchyma.

**Figure 1:**
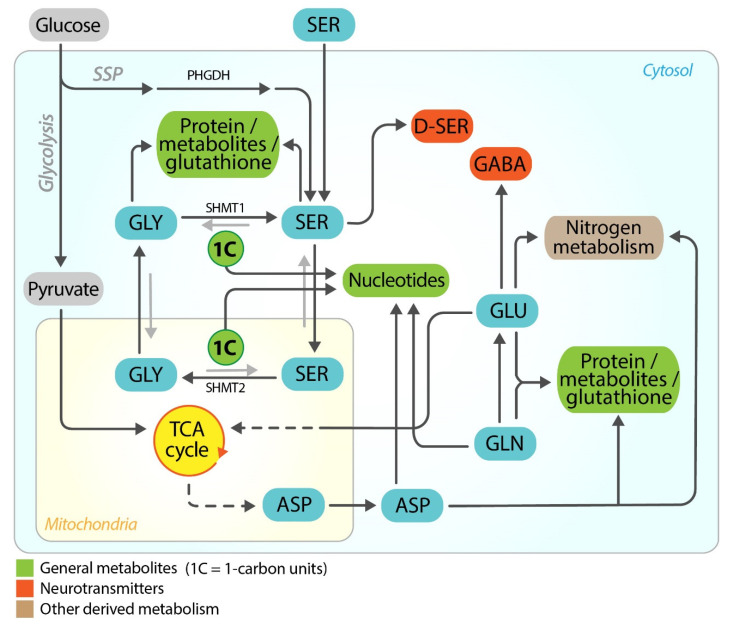
General metabolism of serine, glycine, glutamate, and glutamine. Overview of the possible metabolic pathways relevant to anabolism, redox and nitrogen homeostasis, and energy production. Metabolites specific to the brain, relevant to this review, are also included in red. For clarity, only two enzymes are reported: phosphoglycerate dehydrogenase (PHGDH), responsible for the serine synthesis pathway (SSP), a branch of glycolysis and serine hydroxymethyltransferase (SHMT), responsible for the reversible conversion of serine and tetrahydrofolate (THF) into glycine and 5,10-methylene-THF; SHMT isoforms are present in mitochondria (SHMT2) and cytosol (SHMT1), respectively. These enzymes fuel one carbon metabolism (1C in the figure), which is closely connected to the folate and methionine cycles. Glutamine also contributes to the production of purines, pyrimidines and amino sugars, as well as of nicotinamide adenine dinucleotide phosphate (NADPH, not shown) [28,29], which, in turn, is used to maintain glutathione in its reduced state, thus keeping oxidative stress under control. As a carbon source, glutamine plays a fundamental role in mitochondrial metabolism, supplying the TCA cycle with anaplerotic carbons, accounting for ATP and macromolecules synthesis. The indications “Protein” refer to protein biosynthesis. Serine-derived metabolites include sphingosine and phosphoserine; glycine-derived metabolites include bile acids, heme and creatine; glutamate-derived metabolites include amino acids and polyglutamylate folates (only most relevant metabolites are listed, not necessarily populated in the same cell).

Indeed, these four metabolites are at the crossroad of many anabolic processes in all the cells, being relevant for energy production, nucleotide biosynthesis, nitrogen and redox homeostasis, and neurotransmitter and tissue-specific metabolites (Figure 1). Moreover, this amino acid quartet is also relevant for cancer-specific metabolism. Serine and glycine fuel one carbon metabolism (SGOC) and play a pivotal role in cancer cells, by providing building blocks (molecular components used for the synthesis of nucleotides, lipids, and proteins) and reducing power, as well as the methylation potential necessary to maintain high rates of proliferation [25]. Similarly, glutamine sustains proliferation in many tumor types, by directly contributing to nucleotides biosynthesis (Figure 1) [26,27].

The process of efflux and influx of molecules through the BBB is finely regulated by different groups of transporters with a specifically polarized distribution on endothelial cells. Highly specific transporters facilitate the influx of nutrients down their concentration gradient across the physical barrier of the BBB into the brain parenchyma. On the other hand, Na^+^-dependent-specific transporters are expressed on the abluminal surface to transport metabolites from the brain parenchyma inside the endothelial cells; at this level, the amino acids, through the facilitated transport system expressed on the luminal side, are released into the blood [28]. Through these transporters, for example, amino acids are released into the blood at the BBB level in the process called brain-to-blood efflux. It must be considered that the mechanism is however more complex than as just described, given that the net efflux of amino acids from the brain to the blood is also linked to numerous other chemical-physical factors such as (1) the difference in amino acid concentration between the various compartments, (2) the relative concentrations of the different amino acids which are also linked to the ability of the transporters to mobilize more molecules of the same family, (3) the membrane voltage generated inside the cells (considering that many transporters work using the electrogenic force). To add complexity to the regulatory mechanisms involved, it is also necessary to consider that the amino acid concentration can be regulated for example by forming peptides which thus modify the equilibrium and that the membrane voltage can also be regulated by changes in pH or by the pKa of solutes inside the cell and so on (for a detailed analysis of the brain-to-blood efflux mechanism related to amino acids we suggest [30]). This transport system is part of a mechanism whose goal is to maintain, in the brain extracellular fluid, the optimal concentration of neurotransmitters and amino acids such as glutamate, glutamine, glycine, serine, and others.

**Figure 2:**
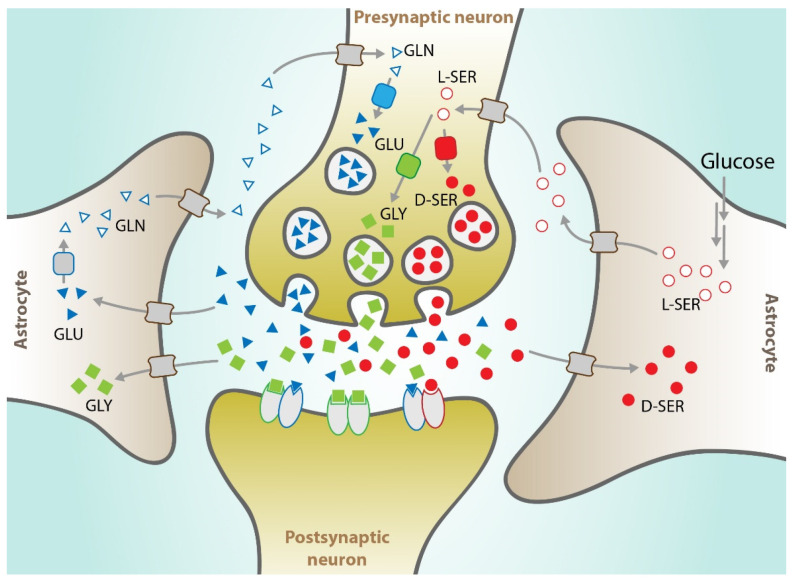
Possible trafficking of selected amino acids at the tripartite synapse. L-serine (empty red circle) is synthesized and released by astrocytes and imported into neurons, to sustain D-serine biosynthesis by serine racemase (in red). D-serine is released and, once in the synaptic cleft, binds to synaptic NMDAR. Glycine (green squares) may also be released by neurons and could activate synaptic NMDARs to a lesser degree than D-serine. D-serine is finally removed from the synaptic cleft by the asc-1 transporters. Glycine is an obligatory co-agonist of glutamate (blue triangles) to activate NMDAR. Many inhibitory neurons release both glycine and GABA, which may act as co-agonists of GlyRs (not shown). GlyT transporter, found mainly in astrocytes, controls glycine concentration. Neuronal glutamate pool is controlled by glutamine released by astrocytes (empty triangles).

As already mentioned, the brain represents a very special microenvironment in terms of trafficking and metabolism of these amino acids as summarized in Figure 2.

Neurons, for example, lacking phosphoglycerate dehydrogenase (PHGDH) [31,32], show a nonfunctional de novo SSP and therefore need a continuous L-serine supply from external sources, such as supporting cells and/or the circulation [33,34]. Astrocytes selectively express the PHGDH enzyme and seem to represent, in the brain, the main source of L-serine that is produced at high levels and exported in the extracellular environment. Extracellular L-serine is reabsorbed by neurons and, through the serine racemase activity, converted into D-serine, which is then released and acts as a neurotransmitter by activating the NMDA receptors [35,36,37]. Glycine is also continuously released in the extracellular brain microenvironment and acts as an inhibitory neurotransmitter by activating different receptors such as the strychnine-sensitive glycine receptor (GlyRs) that mediates the production of a post-synaptic inhibitory potential. The termination of the glycinergic signal is mediated by the re-uptake of glycine into the glycinergic terminations and surrounding glial cells. This process is mainly mediated by the specific Na(+)/Cl(−)-dependent transporters GlyT1 and GlyT2. GlyT1 seems also involved in the regulation of glycine concentration at the level of the excitatory synapses containing N-methyl-D-aspartate receptors (NMDAR) with glutamate and glycine acting as co-agonists. Finally, it was also observed that, in specific areas of the brain, glycine is released together with gamma-aminobutyric acid (GABA) potentiating its inhibitory signal [38,39].

The brain microenvironment is also rich in glutamine and glutamate, which play important functions in the brain and participate in tightly controlling synaptic excitability, a process with high energy demands. While glucose is used as primary energy substrate in the brain, blood-derived glutamine (the most abundant amino acid in the blood) is at the crossroad between central metabolism and neurotransmission [40]. Furthermore, glutamine is a precursor of glutamate, the most abundant amino acid in the brain. In the brain, glutamine/glutamate metabolism occurs through a particular cycle that involves the release and reuptake of the two metabolites by different cells. This complex mechanism requires a peculiar structure called “tripartite synapse” in which the structure formed by the presynaptic and postsynaptic neurons is associated with astrocytes that continuously reabsorb the secreted glutamate to avoid an erroneous and constitutive hyperpolarization of neurons (Figure 2). The glutamate reabsorbed by astrocytes is transformed by glutamine synthase into glutamine that is secreted into the microenvironment and internalized by neurons in this nonexcitatory form. In the presynaptic neurons, glutamine is broken down again into ammonia and glutamate which is packaged in secretory vesicles ready to be released and restart the cycle [22]. Some studies have shown that cell lines of different origins such as breast, prostate, melanoma, and glioma release glutamate into the microenvironment, showing a behavior similar to that of neurons [41,42].

## 4. Serine/Glycine and Glutamate/Glutamine in Brain Metastasis

Triple negative MDA-MB-231 breast cancer cells represent an interesting model to study the involvement of SGOC metabolism in the brain metastasis process. A series of cell lines were obtained from the parental one, each of which, when injected into immunocompromised mice, shows a specific metastatic tropism, in particular for the lung (MDA-MB-231L), for the bones (MDA-MB-231Bo), and for the brain (MDA-MB-231Br). A very recent study revealed that MDA-MB-231Br and MDA-MB-231Bo cells consume purines faster than parental cells. The phenomenon seems to be due to an upregulation of the one carbon metabolism-related proteins serine hydroxymethyltransferase 2 (SHMT2), methylenetetrahydrofolate dehydrogenase 2 (MTHFD2), and its mitochondrial isoform MTHFD1L. Metabolic flux analysis clarified that consumption of mitochondrial serine, used in parental cells to produce ATP and GTP, is significantly more active in metastasizing cells, as suggested by the increased sensitivity of these cells to the inhibition of SHMT proteins with respect to the parental cells. Although the data refers only to MDA-MB-231L cells, the inhibition of SGOC reduces the metastatic potential of these cells [43]. The SHMT1 protein, mainly responsible for the cytoplasmic conversion of glycine to serine, does not appear to be generally overexpressed in brain metastasis deriving from breast cancer; however, a specific shmt1 expressing subtype has been recently identified, showing a dramatically negative association with the overall survival (*p* = 0.002), strikingly suggesting that adaptation to cytoplasmic serine production confers a key advantage for these cells [44]. Interestingly, in the same paper it has been shown that stromal cells in brain metastasis overexpress the SSP enzymes PHGDH, phosphoserine aminotransferase (PSAT), and phosphoserine phosphatase (PSP) as well as SHMT1, suggesting that an increased amount of serine and glycine could be produced and released by the stroma to support cancer cells [44].

Glycine also plays a fundamental role in supporting the development of brain metastases in part due to its involvement in glutathione (GSH) production, the demand of which is increased in breast cancer cells metastasizing in the brain [45]. These cells, to balance the increased levels of reactive oxygen species (ROS) due to an increased respiration, overexpress genes such as glutathione reductase and glutathione S-transferase; the metastasizing cells are therefore up to 60 times more resistant to oxidative stress induced for example by chemotherapy drugs such as bortezomib than parental cells [46]. Glycine is not only employed by brain metastasizing cells to produce GSH. Experiments performed by injecting labeled glucose into patients just before glioma resection show that a substantial part of the labeled glucose is used, in the tumor mass, for the de novo production of glycine through the SSP [47]. Very similar data were observed when analyzing the brain metastasis of a patient affected by breast cancer [47], a detail of particular interest because breast cancer and melanoma cells overexpress some genes of the SSP, as PHGDH, amplified at the genomic level in a specific ER-negative tumor subset. In this model, SSP inhibition induces not so much a drop in serine levels, but a substantial reduction in the flow of α-ketoglutarate from glycine which supports the energy needs of these cells [48]. It has also been reported that, in vivo, the genetic suppression and pharmacologic inhibition of PHGDH attenuated brain metastasis, and improved overall survival in mice [49].

The close connection between neurons and cancer cells has been demonstrated not only in brain metastasis but also in primary tumors [50]. Neurons that innervate tumors can in principle supply nutrients to poorly perfused areas functioning as a surrogate for blood vessels. While the axons are located in the nutrient-poor tumor, the neuronal cell body has access to high levels of nutrients in the circulation. One example is provided by the axons that considerably innervate pancreatic tumor masses. A subset of human pancreatic ductal adenocarcinoma (PDAC) cell lines not expressing the SSP enzymes are dependent on exogenous serine to grow. Pancreatic cells are auxotrophic for serine which is constantly released, even if at low levels, by neurons that innervate the mass [50]. Without the supply of serine from neurons, tumor cells show an inhibition in cell proliferation and respiration due to a reduction in mRNA translation efficiency induced by a stall of the ribosomes on 2 of the six possible serine codons (TCT and TCC), thereby limiting the translation of proteins linked to specific pathways in which the sequences are enriched with TCT and TCC codons. Not by chance, the production of NGF, responsible for innervating the tumor masses, is scarcely affected by the lack of serine. The inhibition of NGF signaling inhibits the formation of innervations responsible for the release of serine even if it is not clear what the real source of NGF is within the tumor mass [50]. Besides serine, it has been also shown that neurons can affect PDAC growth by releasing neurotrophic factors and neurotransmitters [51,52]. Therefore, the axonal–cancer metabolic crosstalk is a critical adaptation to support tumor growth in nutrient poor environments.

Other studies on MDA-MB-231Br and MDA-MB-361 cell lines, metastasizing in the brain the first (see above) and derived from brain metastases the second, show an increased ability to use glutamine in conditions of reduced availability of glucose compared to parental MDA-MB-231 cells. Glutamine appears to be partially converted to glutamate and then to α-ketoglutarate for energy production through the TCA cycle. Glutamine is also invested in the gluconeogenesis process (probably to fuel ribose production), driven by the upregulation of fructose-1,6-biphosphatase on which the cells are completely dependent [53].

In addition to the metabolic one, a new role for glutamate has recently been demonstrated, through the activation of the NMDAR receptor. The autocrine secretion of glutamate stimulates the NMDAR-GKAP signaling axis, where GKAP is a scaffold protein of NMDAR, which activates a cell survival signal and evokes an invasive tumor growth in a mouse model of pancreatic tumor (PanNET) [54]. Interestingly, it has been shown that MDA-MB-231Br or mouse TS1 breast cancer cell lines, when injected into a mouse, acquire the ability to produce the astrocytic “pseudo-tripartite synapse”. Cancer cells, through the pseudo-tripartite synapses established between metastatic cells and glutamatergic neurons, “capture” part of the glutamate secreted by neurons to activate the survival signal through the NMDAR receptor. This paracrine supply of glutamate has been considered to support the “seed and soil” theory of organ-selective metastasis [55].

The metabotropic glutamate receptors (mGluRs), known for their roles in synaptic signaling, seem to also play a fundamental role in the metastatic process. Recently, an aberrant glutamate signaling has been shown to participate in the transformation and sustenance of several cancer types that form metastases in the brain including melanoma and breast cancer. Glutamate secreted by cancer cells induces excitotoxicity in surrounding neurons creating space for the expansion of the growing tumor [56]. mGluRs have been shown to be overexpressed in melanoma and breast tumors [57,58], and to be involved in late steps of cancer progression such as the ability of certain cancer cells to survive even in the absence of an extracellular matrix that guarantees their anchorage (anchorage-independent growth) [59]. mGluRs are also involved in brain-specific metastasis. Inhibition of glutamate utilization was able to reduce the growth of melanoma metastases in the brain [56,57] and to reduce in vitro and in vivo tumor progression by inducing apoptosis in breast cancer [60]. Elevated levels of extracellular glutamate were detected in the conditioned medium of melanoma cells that show enhanced metastatic potential [61]. MGluR1 has been also involved, in metastatic cells, in an angiogenic mechanism, which results in larger tumor growth in vivo. Glutamate may mainly modulate the development of metastasis in the brain partly by increasing angiogenesis, and mGLUR1 inhibition reduces in vivo micro vessel density [60], indicating that suppression of the double role played by glutamate signaling in metastases initiation and survival could be a promising anti-angiogenic tool and a potential target in treatment of hemorrhagic brain metastases [57].

## 5. Importance of Amino Acids in Brain Parenchyma Invasion

Extracellular amino acids are recently emerging as fundamental players not only in supporting the growth of metastatic cells in the brain parenchyma, but also in the process of extravasation and invasion [58,62,63]. A very recently published work has shown that the bioavailability of asparagine is also fundamental in the development of metastases, demonstrating that asparagine synthase is significantly overexpressed in a particular model of highly metastasizing cells and in secondary lesions of patients with breast cancer with metastases to bone, brain, and lungs [62]. The decrease of bio-available asparagine dramatically reduces the invasive ability of these cells in vitro and the ability to form metastases in vivo, showing, apparently, no effect on the formation of the primary tumors. Although the mechanism has not been completely clarified, it seems that several proteins, upregulated by the activation of the epithelial to mesenchymal transition mechanism, are enriched in asparagine residues and that therefore the bioavailability of this amino acid is fundamental in their production [62].

**Figure 3:**
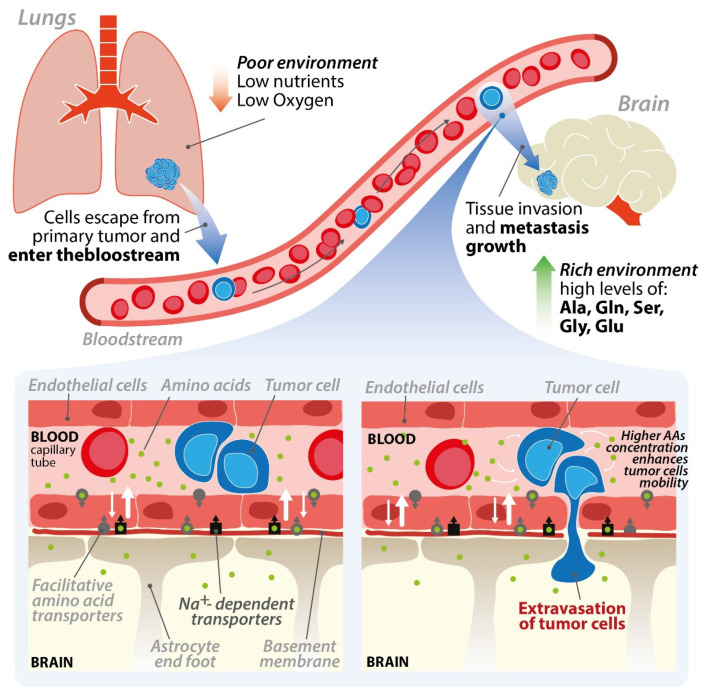
Hypothetical involvement of the brain to blood efflux in the metastatic process (schematic representation) (modified from [63]). An increased number of amino acids in the brain microvasculature stimulate the chemo-kinetic ability of metastatic cancer cells triggering the brain parenchyma invasion.

We have recently shown that serine and glycine are able to stimulate the chemo-kinesis of lung cancer cells through a mechanism involving SHMT1-dependent control of cytoplasmic serine, which increases ATP production and reduces ROS formation. Reducing the import of serine and glycine activates AMP kinase, determining a complete arrest of cell migration, and possibly affecting invasion of brain parenchyma [63]. Our working model is based on the concept of brain-to-blood efflux (Figure 3).

In the brain, the amount of amino acids in the extracellular fluid must be kept low to avoid unwanted stimuli or the activation of toxic mechanisms for the cells [64]. As previously mentioned, neurotransmitters and amino acids are rapidly reabsorbed by the cells in the microenvironment through specific transporters which are also expressed on endothelial cells and participate in the blood-to-brain efflux mechanism [65]. The sodium-dependent transporters of the ASC family (SLC1A4 and SLC1A5), for example, are responsible for efflux of alanine, serine, and cysteine, while the excitatory amino acid transporter (EAAT) family controls glutamate efflux [66] and the sodium neutral amino acid transporter 3 (SNAT3) is involved in alanine, proline, histidine, serine, and asparagine efflux [30,66]. It is known that cancer cells are able to reach the microvasculature of the brain and adhere to it, often forming micro-clots. We hypothesize that the slowing of blood flow in the event of a micro-clot, associated with the release of amino acids through the brain-to-blood efflux mechanism, can increase the local concentration of amino acids to a sufficient level to trigger the invasion process by increasing the chemo-kinetic ability of cancer cells [63].

Another hypothesis on the mechanisms that trigger the migration of disseminating cancer cells in the brain parenchyma is related to the ability of these cells to damage the endothelium of the BBB, altering the integrity of the barrier, thus releasing into the blood the molecules, including amino acids, contained in the BEF, further stimulating invasive mechanisms [67]. For example, it has been reported that serine proteases released by metastatic melanoma cell lines A2058 and B16/F10 damage the integrity of the barrier by inducing apoptosis of endothelial cells [68]. Other proteases, such as urokinase-type plasminogen activator (uPA), or seprase or matrix metalloproteinases (MMPs) released by melanoma cells, are able to damage tight junctions among the endothelial cells, altering the permeability of the BBB [67]. Moreover, metastatic cells can also alter the metabolism of the endothelial cells in the brain microvasculature for example inhibiting the expression of the endothelial cell fatty acid transporter Mfsd2a inducing, also in this case, the leakage of the BBB [69]. We have recently shown that BEF is able to dramatically stimulate the chemo-kinesis of lung cancer cell lines and that the amino acids serine and glycine play a fundamental role in this process [63]. From the clinical point of view, inhibitors of the import of these amino acids may offer novel therapeutic opportunities to control the extravasation process, dramatically reducing brain metastasis formation.

## 6. Conclusions

Brain metastases have devastating effects on the life expectancy of patients, in particular for those affected by melanoma, lung, or breast cancer. In the last few years, the role of amino acids in the processes of extravasation and survival of cancer cells in the brain microenvironment is becoming evident. The new data produced are demonstrating that an innovative therapeutic approach, based on the limitation of the availability of amino acids in the serum of the patients, or on the inhibition of their uptake by cancer cells, could represent a successful and paradigm-shifting change in the approach to this fundamental issue.

## Abbreviations

CXCR-4	C-X-C chemokine receptor type 4
CCR7	C-C chemokine receptor type 7
CXCL12/SDF-1α	stromal cell-derived factor 1
CCL21/6Ckine	C-C chemokine ligand 21
BBB	blood–brain barrier
SSP	serine synthesis pathway
BEF	brain extracellular fluid
SGOC	serine and glycine one carbon metabolism
PHGDH	phosphoglycerate dehydrogenase
GlyR	strychnine-sensitive glycine receptor
GlyT1	glycine transporter 1
GlyT2	glycine transporter 2
NMDR	N-methyl-D-aspartate receptors
GABA	gamma-aminobutyric acid
SHMT2	serine hydroxymethyltransferase 2
MTHFD2	methylenetetrahydrofolate dehydrogenase 2
MTHFD1L	methylenetetrahydrofolate dehydrogenase 1L
PSAT	phosphoserine aminotransferase
PSP	phosphoserine phosphatase
GSH	glutathione
ROS	reactive oxygen species
PDAC	pancreatic ductal adenocarcinoma
mGluRs	metabotropic glutamate receptors
SLC1A4	solute carrier 1A4
SLC1A5	solute carrier 1A5
EAAT	excitatory amino acid transporter
SNAT3	sodium neutral amino acid transporter 3
THF	tetrahydrofolate
SHMT1	serine hydroxymethyltransferase 1
NADPH	nicotinamide adenine dinucleotide phosphate
TCA	tricarboxylic acid cycle
